# The effects of alcohol and co-witness information on memory reports: a field study

**DOI:** 10.1007/s00213-022-06179-5

**Published:** 2022-06-28

**Authors:** Georgina Bartlett, Ian P. Albery, Daniel Frings, Julie Gawrylowicz

**Affiliations:** 1 Centre for Addictive Behaviours Research, Division of Psychology, London South Bank University, 103 Borough Road, London, UK; 2 Division of Psychology and Forensic Sciences, Abertay University, Dundee, UK

**Keywords:** Eyewitness memory, Intoxication, Memory conformity, Source monitoring

## Abstract

**Rationale:**

Witnesses who discuss a crime together may report details that they did not see themselves but heard about from their co-witness. Co-witness information may have beneficial and harmful effects on memory accuracy depending on whether the information was correct or incorrect.

**Objectives:**

Given the prevalence of intoxicated witnesses, it is imperative to understand how alcohol influences this effect.

**Methods:**

The present study asked pubgoers (*n* = 67) at varying levels of intoxication to recall a mock crime video after having also watched a video witness statement containing both correct and false information.

**Results:**

Increased intoxication was associated with decreased confidence, completeness and accuracy, but no increased tendency to report false information. Exposure to incorrect post-event information (PEI) can lead to the incorporation of incorrect information, whereas exposure to correct PEI increased accuracy, regardless of individuals’ alcohol intoxication status.

**Conclusions:**

Thus, whilst discussion and intoxication can negatively impact eyewitness memory, discussion may also have benefits for both sober and intoxicated witnesses.

Alcohol intoxication in witnesses and victims is common (Crossland et al. 2018; Evans et al. 2009; Monds et al. 2021) and such witnesses often play a comparable role to their sober counterparts in criminal investigations (Palmer et al. 2013). Both were just as likely to make a suspect ID and to provide a suspect description. It is therefore imperative to better understand how alcohol impacts memory performance in applied forensic settings. Findings from lab studies using low to moderate doses of alcohol (blood alcohol concentration [BAC] < 0.08%) suggest that acute intoxication may lead participants to produce less complete memory accounts (i.e. recalling fewer correct details overall) without negatively impacting the accuracy of individual’s recall (Bartlett et al. 2021; Flowe et al. 2016; Hagsand et al. 2017). Intoxicated participants also appear to be less confident in their recollections compared to sober controls (Crossland et al. 2016; Flowe et al. 2017). The majority of lab studies did not find alcohol-related differences in individual’s suggestibility using misinformation paradigms (Bartlett et al. 2021; Flowe et al. 2019; Thorley & Christiansen 2018) or the Gudjonsson Suggestibility Scale (Mindthoff et al. 2021). Evans et al. (2019) found that intoxicated participants were only more vulnerable to incorrect suggestions when tested after a delay.

Whilst intoxication may affect one’s memory recall, it is important to consider its impact on metacognition too. Gawrylowicz et al. (2019) found that intoxicated individuals were less likely to use ‘don’t know’ responses to screen out incorrect responses to unanswerable questions. Little evidence for metacogntive differences was found by Evans et al. (2017). Only for the recognition task were sober individuals slightly better at discriminating accurate from inaccurate responses by using confidence judgements. Flowe et al. (2019) did not find alcohol-related differences in confidence-accuracy calibrations.

In line with lab research, field studies employing higher BACs (> 0.09%) showed that accounts by severely intoxicated indivdiuals are less complete than those produced by sober ones (Altman et al. 2018; Altman et al. 2018; Crossland et al. 2016). In contrast to low to moderate intoxication levels, high levels may also negatively impact recall accuracy, that is, as alcohol levels increase, the proportion of accurate details recalled decreases (Altman et al. 2018; 2019; Van Oorsouw et al. 2015) and the number of ‘don’t know’ responses increases (Crossland et al. 2016). Similarly, fieldwork testing immediate and delayed suggestibility showed that as intoxication levels increase, so does one’s willingness to go along with incorrect suggestions (Van Oorsouw et al. 2015; 2019). Together, this work suggests that lower doses of acohol may reduce the recollection of details without negatively affecting recall accuracy or one’s susceptibility to misinformation. However, higher doses of alcohol may lead to lower completeness and sometimes accuracy as well as increased suggestibility in witnesses.

The presence of a co-witness may impact also the reliability of eyewitness evidence. Skagerberg and Wright (2008) reported that 88% of their surveyed witnesses had a co-witness, and of those 58% reported discussing the crime with one another. This highlights that witnesses may report information they did not observe but obtained through discussion with fellow witnesses, a phenomenon called memory conformity (Gabbert et al. 2003; Ito et al 2019). Laboratory studies have examined the effects of co-witness discussion on autobiographical memory reports (Gabbert et al. 2003; Paterson et al. 2012; see Hope & Gabbert 2019) and found that recall of an event can be strongly influenced by another person’s account. The types of stimuli used varies considerably, including mock crime videos (Paterson et al. 2009), picture slideshows (Goodwin et al. 2013) and confederate accounts (Roediger et al. 2001).

Few studies have examined the effect of acute intoxication on memory conformity. Thorley and Christiansen (2018) tested drunk, sober and placebo participants’ susceptibility to concur with the suggestions of a seemingly drunk confederate during a collaborative recall task. All participants reported contagion items regardless of their intoxication state. Also, Bartlett et al. (2021) asked intoxicated and sober dyads to remember and discuss a mock-crime. Unknown to participants, each dyad member saw a slightly different version of the crime including unique details not present in the other version. After discussion, intoxicated and sober dyads were equally likely to report misleading information in their individual recall. It should be noted that intoxication levels were relatively low in both studies (0.06% BAC) and elevated levels could yield different results.

Most work examining how alcohol affects the tendency to report misinformation has used leading questions (Van Oorsouw et al. 2015; 2019) or false information incorporated into written or oral accounts (Flowe et al. 2019; Schreiber Compo et al. 2012; Thorley & Christiansen 2018). Work by Evans et al. (2019) incorporated written misinformation in the form of a forced-choice recognition test that contained answers that had already been circled, seemingly by a previous participant. Whilst this work highlights the risks associated with exposure to misinformation, it can sometimes have benefits, i.e. when post-event information (PEI) is correct. For example, Paterson and Kemp (2006) introduced correct and incorrect PEI via different sources (co-witness information vs. leading vs. media reports). Whilst observing the typical misinformation effect, they also found that participants who were exposed to correct PEI were significantly more accurate than those who did not receive any PEI. Likewise, Harkness et al. (2015) found that exposure to correct and incorrect PEI through confederate discussion, after participants engaged in an ego-depletion or a control task, increased both the number of misinformation items recalled and the number of correct PEI reported. Interestingly, ego-depleted participants incorporated more misleading and less accurate PEI. To date, the impact of alcohol on potential positive effects of witness discussion has not been tested.

This is the first field study to examine the tendency of sober and intoxicated mock-witnesses to incorporate misleading and correct PEI from a sober co-witness. After watching a video of a mock-crime, bar patrons viewed a video of a witness reading a prepared statement containing correct and erroneous details. Presentation of PEI via a video witness was used to ensure that the source of PEI was always sober and because engaging in a ‘live’ discussion could be problematic in a field setting. It was hypothesised that intoxication would significantly negatively predict recall accuracy, completeness and participants’ confidence judgements (see Altman et al. 2018; Crossland et al. 2016; Jores et al. 2019 [meta-analysis]). We also hypothesised that participants would incorporate both correct and incorrect PEI in their recall (see Harkness et al. 2015; Paterson & Kemp 2006) and that the tendency to report both correct and incorrect PEI would increase with increasing intoxication (Van Oorsouw et al. 2015; 2019).

## Method

### Participants

Sixty-seven participants were sampled opportunistically during the course of data collection. The sample comprised 36 males and 26 females, with five participants choosing not to state their gender (mean age = 33.4 years, *SD* = 11.90, range: 18–65). An achieved power analysis was conducted to establish the power of the analysis with the smallest sample size (66). It indicated that a sample of 66, with three groups and one covariate and an effect size of *f* = 0.38, achieved a power of 0.86.

### Materials

#### Videos

Participants were presented with two videos created for the present study. The first, lasting 2 min and 10 s, depicted a mock crime occurring at a pub. The video showed a woman entering a pub and ordering a drink at the bar from a female bartender. After a few minutes, she left the pub to have a phone conversation on her mobile. She left her bag on one of the barstools. A second female enters and sits next to the bag. She then rummages through the bag and steals several items from it. After the perpetrator left, the victim re-emerged and realised that she has been mugged. The second video was 58 s long and showed a witness to the incident reading their statement to the camera. This witness provided four pieces of accurate information (e.g. the victim had a black bag when in fact the bag was black) and four pieces of inaccurate information (e.g. the barstools were green when in fact the stools were red). Participants were only informed that the video they were watching depicts a witness giving their statement about the incident that they just watched.

#### Free and cued recall test

The study included free recall and cued recall components. In the free recall, participants were asked to recall the mock-crime in as much detail as they could remember. Subsequently, twelve cued recall questions tested participants’ memory for specific items including events, details of people involved and details about surroundings. Of these questions, four pertained to erroneous PEI participants received from the video witness, four to correct PEI and four related to details for which participants had not received any kind of re-exposure. For each question, participants were asked to indicate their confidence in their answer on a five-point Likert scale, ranging from one (not confident at all) to five (very confident). A single source monitoring question at the end of the cued recall required participants to determine whether their responses came from their own memory of the event, the co-witness or both. Scoring procedures for these tests are reported in the “Results” section.

#### Drinking behaviour

The AUDIT-C (Bush et al. 1998) was used to measure regular drinking behaviour, a shortened version of the full AUDIT screening that identifies risky drinking behaviours by measuring alcohol consumption. The AUDIT-C is scored from 0 to 12, with scores of above 7 indicating potentially problematic alcohol consumption. Participants were also asked to report how many alcoholic drinks they had consumed in their current session of drinking and how intoxicated they felt.

#### Alcohol intoxication

A Lion Alcometer 500 breathalyser was used to determine breath alcohol content (approximately 15 min after the study began, following the main measures). Participants were asked not to consume any drinks during the study.

### Design

The study used a mixed design with confederate information as a within subject factor (3 levels: correct, incorrect, no information). Participant intoxication was the predictor variable. Dependent variables were memory accuracy, completeness, confidence judgements and PEI reported. The study received ethical approval at the University ethics panel at London South Bank University (ethical approval number SAS1823).

### Procedure

Potential participants were approached in two pubs in Berkshire and Dorset (locations redacted for blind review). Neither pub was affiliated with a university and attracted a range of patrons including both students and working adults. Data was collected between 2 and 8 pm to reduce the likelihood of encountering those who may be too intoxicated to consent. Two researchers were present during each testing session and would make a joint decision as to whom to approach. Pubgoers were not approached and asked to take part if they were visibly intoxicated, e.g. slurring their words or unsteady on their feet. With the consent of pub licensees, participants were approached by the researcher and asked if they would be interested in taking part in a study on the effect of alcohol on eyewitness memory. Upon consent, participants were taken to a quiet area of the pub to complete the study individually on a laptop. Participants were first instructed to read a participant information sheet and were asked by the researcher if they understood the information or had any questions prior to proceeding. Participants were informed that they would be watching a video of a mock crime and would have to answer some questions. They were provided with headphones to watch the video of the crime taking place and the video of the witness reading her statement (in that order) after which they engaged in an unrelated ‘spot the difference’ filler task for 10 min. Participants were then asked to complete the free recall task and then the cued recall questions in written format. After completing these elements, participants completed the AUDIT-C and were breathalysed. Finally, participants were fully debriefed and thanked for their time.

## Results

### Data scoring

An a priori scoring sheet was used to score participants’ free and cued recall responses (see Van Oorsouw et al. 2012; 2015). The sheet contained 53 details that referred to the surroundings, events and people depicted in the video.^1^ The scoring sheet outlined the details of the video in their smallest units of description (e.g. barstool with orange cushion and brown legs represented 5 units of information: *Barstool (1) with orange (1) cushion and (1) brown (1) legs (1)).*

The free recall data were scored according to whether each detail participants reported was correct, an error, unscorable information or incorrect PEI. A detail was scored as correct if it accurately described the events in the video (e.g. ‘the walls in the pub were red’ when indeed the walls were red). Participants received a correct point for each unit of correct information provided (e.g. ‘the victim was a dark-haired female in a black and white dress’ would be scored as four correct details, i.e. *dark-haired (1), female (1) and black and white (1) dress (1)*). A detail was described as an error if it incorrectly described a detail from the video (e.g. ‘the victim had blonde hair’ when in fact the victim had dark brown hair). Information was unscorable when it referred to subjective feelings or the opinions of participants (e.g. ‘I think she looked shifty’). Finally, incorrect PEI referred to erroneous details reported by the video confederate that participants incorporated into their own accounts.

For the free recall data, the total number of details in each response category was recorded. Additionally, an accuracy rate was computed by dividing the number of correct details by the total number of details reported. A subset of fourteen (> 20% of the total sample) free recall accounts was independently coded by a second individual who was blind to the BACs of participants and the hypotheses of the study. Significant inter-coder reliability was shown across all response categories (see Table 1). For the cued recall data, each question was scored as either ‘correct’, ‘incorrect’, ‘incorrect PEI’ or ‘I don’t know’. Twenty percent of cued recall data was similarly double scored, and significant inter-coder reliability was also demonstrated (see Table 2). Accuracy rates were computed for each question category (questions for which participants received correct PEI, incorrect PEI or no PEI) in the same way as outlined above. Both coders were blind to participant intoxication level at the point of coding the data. The second coder was additionally blind to the study hypotheses.

**Table 1:** Intra class correlation coefficients between coders for each response type in the free recall

*Response type*	*ICC*	*p*	*95% CI*
Correct	0.98	< 0.001	0.95, 0.99
Error	0.85	0.001	0.53, 0.95
Misinformation	0.95	< 0.001	0.85, 0.98

**Table 2:** Intra class correlation coefficients between coders for each response type in the cued recall

*Response type*	*ICC*	*p*	*95% CI*
Correct	0.99	< 0.001	0.98, 0.99
Error	0.98	< 0.001	0.94, 0.99
Misinformation	1.00		1.00, 1.00
I don’t know	1.00		1.00, 1.00

For the source monitoring question, responses were scored as incorrect when (a) participants incorporated incorrect PEI from the video witness in their cued recall answers but incorrectly stated that they only included answers based upon the video and/or (b) if participants did not include any incorrect PEI but incorrectly stated that they did include details based upon the witness. Responses were scored as correct when participants reported incorrect PEI and stated that their answers were based on the statement by the co-witness or both (i.e. co-witness and video). Responses were also coded as correct if participants did not report any incorrect PEI and stated that their answers were entirely based on their own memory of the video.

### Subjective and objective consumption of alcohol

The mean number of alcoholic drinks participants reported having consumed was 2.45 (*SD* = 2.02) with a range from 0 to 9. The mean BAC of participants who were intoxicated was 0.05%, with a range from 0.01 to 0.19%. The number of drinks participants reported having consumed was significantly positively correlated with their BAC reading, *r* = 0.61, *N* = 67, *p* < 0.001, *95% CI* [0.47, 0.75]. The mean score on the AUDIT-C was 8.13 (*SD* = 2.26), ranging from 4 to 12. Bivariate correlations indicated a significant positive relationship between AUDIT-C score and the number of drinks consumed on the night of testing, *r* = 0.36, *p* = 0.003. Additionally, there was a significant positive relationship between BAC and AUDIT-C score, *r* = 0.30, *p* = 0.012. Those who scored higher on the AUDIT-C reported consuming more alcoholic beverages on the testing night and had a higher BAC. Participants were asked what type of alcoholic drink, if any, they had consumed prior to testing. Twenty-two participants reported consuming beer, three reported wine, nine spirits and seven reported consuming a mix of beverages. The remaining twenty-six participants either did not consume any alcoholic drinks or did not report what type of drinks they had consumed.

### Free recall

For the free recall data, correlations with bootstrapping of 1000 samples were used to examine the relationship between intoxication level and memory completeness, accuracy and the number of incorrect PEI items reported. Memory completeness was calculated as the total number of details participants reported, whilst accuracy rate was calculated as the number of correct details reported divided by the total number of details reported. There were significant negative correlations between BAC and completeness, *r* =  − 0.48, *N* = 63, *p* = 0.01, *95% CI* [− 0.64, − 0.25], and intoxication level and accuracy rate, *r* =  − 0.64, *N* = 63, *p* < 0.001, *95% CI [*− 0.83, − 0.20]. Sixteen percent of participants included at least one piece of misinformation in their free recall accounts, although there was no significant correlation between intoxication level and amount of misinformation reported, *r* =  − 0.09, *N* = 67, *p* = 0.484, *95% CI [− 0.27, 0.15].* This suggests that increased intoxication levels were related to reduced accuracy and less complete accounts but did not make participants more vulnerable to incorporate misinformation in their free recall accounts.

### Cued recall

Analyses of cued recall data examined how alcohol and the video co-witness influenced participants’ recall accuracy, completeness, confidence in their responses and their ability to monitor the source of the reported information.

#### Recall completeness

There was a significant positive correlation between intoxication level and the number of ‘I don’t know’ responses given in the cued recall, *r* = 0.45, *N* = 67, *p* < 0.001, *95% CI* [0.09, 0.68]. Participants responded with ‘I don’t know’ more frequently when they were more intoxicated.

There was a significant, negative correlation between intoxication level and the number of correct details reported in the cued recall, *r* =  − 0.65, *N* = 67, *p* =  < 0.001. As intoxication increased, the number of correct details reported decreased.

#### Confederate influence and incorrect PEI

Thirty-three percent of participants reported at least one piece of incorrect PEI in their cued recall (*M* = 1.32, *SD* = 0.5). There was no significant correlation between intoxication level and the amount of incorrect PEI reported, *r* =  − 0.06, *N* = 67, *p* = *0*.624, *95% CI* [− 0.26, 0.20]. That is, participants with elevated intoxication levels did not incorporate more misinformation in their cued recall than those with lower levels.

In order to examine the potential benefits of exposure to correct PEI, accuracy rates were computed for each question category (i.e. questions relating to incorrect PEI from the video witness, correct PEI and no PEI) for each participant. Accuracy rates were computed by dividing the number of accurate responses by the total number of responses for each subset of questions including ‘I don’t know’ responses.

A mixed linear model with bootstrapping of 1000 samples was computed to examine the effect of PEI type (correct, incorrect, no information) and intoxication level on accuracy rate. PEI type was added as a fixed factor and intoxication was added as a fixed covariate in the model. PEI type significantly predicted accuracy rate *F* (2, 192) = 21.64, *p* < 0.001. Participants’ accuracy rates were significantly higher for questions pertaining to correct PEI from the video witness (*EMM* = 0.856, *SE* = 0.03, *95% CI* [0.80, 0.91]) than for questions relating to details where the co-witness had given no information (*EMM* = 0.50, *SE* = 0.03, *95% CI* [0.44, 0.55]). Participants also had significantly higher accuracy rates for questions pertaining to correct PEI than incorrect PEI (*M* = 0.54, *SE* = 0.03, *95% CI* [0.48, 0.59]) (*p*s < 0.001). Participants were not significantly more accurate in response to neutral questions than questions for which they received incorrect PEI (*p* = 0.307). Intoxication significantly negatively predicted accuracy rates for each question category, *F* (1, 192) = 76.25, *p* < 0.001. The interaction between intoxication and PEI type was not significant, *F* (2, 192) = 0.87, *p* = 0.42 (see Table 3 for relevant coefficients and confidence intervals).

**Table 3:** Coefficients and confidence intervals for the effect of intoxication and PEI type on accuracy rate in the cued recall. *Parameters are set to 0 as they are redundant in the model

Information type	*b*	SE	*95% CI*
No PEI	* − 0.03*	0.06	* − 0.16, 0.09*
Correct PEI	*0.28*	0.05	*0.19, 0.37*
Incorrect PEI*	*0*	0	*0*
Intoxication	* − 3.98*	0.58	* − 5.19, − 2.87*
No PEI × intoxication	* − 0.26*	0.91	* − 1.9, 1.63*
Correct PEI × intoxication	*1.03*	1.03	* − 0.71, 3.33*
Incorrect PEI × intoxication*	*0*	0	*0*

Thus, exposure to incorrect PEI may undermine a witness’ recall due to the potential for this incorrect information to be incorporated into one’s own recall, whereas exposure to correct PEI can boost accuracy, regardless of one’s alcohol intoxication status. Alcohol intoxication negatively impacts accuracy during cued recall responding but does not interact with different types of PEI.

### Confidence

A mixed linear model was computed to examine the relationship between intoxication, PEI type and participant confidence, with PEI type as a fixed factor and intoxication as a fixed covariate. PEI type significantly predicted participants’ confidence, *F* (2, 193) = 7.22, *p* = 0.001. Participants were significantly more confident in responses to questions pertaining to correct PEI (*M* = 4.36, *SE* = 0.09, *95% CI* [4.18, 4.54]) than to questions pertaining to no PEI (*M* = 3.78, *SE* = 0.09, *95% CI* [3.60, 3.96]) or questions relating to incorrect PEI (*M* = 3.87, *SE* = 0.09, *95% CI* [3.68, 4.05]) (*ps* < 0.001). Intoxication was also a significant negative predictor of participants’ confidence *F* (1, 193) = 16.57, *p* < 0.001. As intoxication increased, participants’ confidence decreased. There was no significant interaction between PEI type and intoxication on reported confidence levels *F* (2, 193) = 0.63, *p* = 0.532 (see Table 4).

**Table 4:** Coefficients and confidence intervals for the effect of intoxication and PEI type on confidence for the cued recall data. *Parameters are set to 0 as they are redundant in the model

Information type	*b*	SE	*95% CI*
**No PEI**	− 0.21	0.19	− 0.57, 0.19
**Correct PEI**	0.42	0.17	0.08, 0.78
Incorrect PEI*	0	0	0
**Intoxication**	− 7.63	3.55	− 12.80, − 0.59
**No PEI** × **intoxication**	3.79	4.48	− 7.19, 10.79
**Correct PEI** × **intoxication**	2.33	5.45	− 8.58, 12.56
**Incorrect PEI** × *intoxication	0	0	0

### Source-monitoring

In order to examine source-monitoring abilities, participants were asked to indicate whether the information they used in response to the cued recall questions came from their own memory, the co-witness or both. In total, 85.1% of participants reported only using their own memory, whilst 14.9% reported using both their own memory and the co-witness’s statement. None of the participants reported using only the witness’ statement.

It is not possible to state whether those who recalled accurate information relied on the witness’ account or used their own memory of the event. However, incorrect PEI reported by participants could only have been encountered by the co-witness. Therefore, a Pearson’s chi-square analysis was used to examine the association between reporting at least one incorrect PEI detail and answering the source monitoring question incorrectly. There was a significant association between reporting at least one piece of incorrect PEI and answering the source monitoring question incorrectly, *c*^2^ (1) = 38.27, *p* < 0.001. Participants who reported at least one piece of incorrect PEI were significantly more likely to incorrectly state that their responses came from their own memory than to correctly identify that they had used information from the video witness. Odds ratios indicate that participants who did not report any incorrect PEI were 173.5 times more likely to correctly identify the source of the information reported. Additionally, a logistic regression showed no relationship between BAC scores and source-monitoring accuracy, *c*^2^ (1) = 0.03, *p* = 0.863.

## Discussion

This field study examined the impact of acute alcohol intoxication and different types of co-witness PEI on eyewitness recall and memory conformity. It was hypothesised that alcohol intoxication would significantly reduce the accuracy and completeness of and confidence in mock witnesses’ testimony and also increase the tendency of participants to report misinformation. We were also interested in how alcohol impacts source-monitoring abilities, a research question that has been neglected thus far.

In line with previous field studies (Altman et al. 2019; Crossland et al. 2016; Van Oorsouw and Merckelbach 2012; Van Oorsouw et al. 2015; 2019), our findings showed that increased alcohol intoxication was associated with poorer completeness of participants’ memory accounts. Elevated levels of intoxication were also associated with lower accuracy rates. Contrasting earlier fieldwork findings on suggestibility (e.g. Van Oorsouw et al. 2015; 2019), no association was found between intoxication level and the incorporation of misinformation. In agreement with fieldwork by Van Oorsouw and Merckelbach (2012) and Crossland et al. (2016), increased levels of intoxication were associated with decreased confidence judgements in response to cued recall questions. Thus, intoxicated participants’ confidence judgements were a correct appraisal of their performance in the task.

We also found that as intoxication increased, so did ‘I don’t know’ (IDK) responses in the cued recall. This is in line with field research by Altman et al. (2018; 2019) and Crossland et al. (2016) who found that participants’ BAC significantly predicted the use of IDK responses. The increased use of IDK responses by participants with higher BACs could be explained by alcohol-induced deficits at encoding. Elevated BAC levels may lead to less information being encoded and subsequently being transferred to long-term memory. At the same time, high BAC levels may negatively affect an individual’s recall ability. Future alcohol studies would do well to test participants’ memory whilst still intoxicated and after a delay when sober again, to disentangle alcohol-related effects on encoding and recall. Increased IDK responding might be an indication of poor memory of the event. Much like the appraisal of one’s own confidence, using IDK when one’s memory is incomplete or inaccurate is an indication of good metacognitive skill (Evans et al. 2017).

To summarise, in line with previous fieldwork and contrasting the majority of lab studies, we found that acute alcohol intoxication in a real-life bar setting did negatively impact eyewitness memory performance in some ways. As BACs increased, the quantity and quality of recalled information decreased. Individuals with higher BACs were *not* more suggestible but less confident in their answers overall and were more likely to answer IDK.

Whilst witnesses are at risk of reporting erroneous information gained from a co-witness (Paterson et al. 2012), the present study suggests that there may be benefits from memory collaboration (see Vredeveldt et al. 2016; Vredeveldt et al. 2017). Unlike ego depletion (Harkness et al. 2015), acute alcohol intoxication did not lead to a detectable increase in the incorporation of incorrect PEI and/or a decrease in the incorporation of correct PEI.

Correctly identifying the source of retrieved information (e.g. whether an event really took place or was just imagined) is an important metacognitive skill (Johnson et al. 1993). Individuals may go along with erroneous suggestions because they fail to accurately determine the source of their memories. We found that alcohol intoxication *does not* significantly diminish one’s source-monitoring judgement.

### Limitations

This study examined the effect of alcohol and co-witness exposure on eyewitness recall after a brief delay which is not consistent with real-life where there is often a long delay between witnessing a crime and the police interview. Also, because other work suggests that susceptibility to misinformation can increase after a delay (e.g. Van Oorsouw et al. 2015), future research should include longer delays and allow for the testing of the impairing effects of alcohol at encoding and retrieval.

Exposure to co-witness information was operationalised by presenting participants with a statement read out by a video co-witness. Paterson and Kemp (2006) showed that although indirect co-witness exposure (reading a co-witness statement) led to reporting of PEI, direct co-witness discussion (discussing the event with a confederate) was a more influential source. Future field research should examine the effects of alcohol on memory conformity when presented via direct co-witness exposure. Moreover, whilst the correct and incorrect PEI reported by the co-witness all referred to the surroundings and events of the original mock crime, no attempt was made to ensure that the details were directly comparable in terms of salience. As such, it is possible that the low reporting of incorrect PEI is due, in part, to the details being less salient than correct PEI. Future work should ensure that both correct and incorrect PEI is equal in their salience and further study the effect this might have on the tendency to report such details.

The study design was correlational; this was to investigate the relationship between different BAC levels on the outcome variables. However, this approach has limitations. Those that are heavier drinkers may differ from lighter drinkers in more ways than BAC level. Heavier drinkers self-reported more problematic drinking on the AUDIT-C. Previous research has identified that task impairment is influenced by drinking experience (Fillmore and Vogel-Sprott 1996). As such, the effect of BAC on memory reports may also be influenced by these additional factors. However, encountering a range of intoxication levels and drinker types is consistent with a real-life drinking setting. Wall et al. (2000) highlight the importance of context when measuring the effects of alcohol on behaviour and cognition. As such, the study provides useful insight into the effects of alcohol and co-witness information in a field setting.

## Conclusions

Our results suggest that witnesses’ recall completeness and accuracy, and confidence in their memory accounts, are negatively affected by alcohol. Thus, moderately to highly intoxicated witnesses might not only be less reliable but also be perceived as less credible by jurors due to their undermined confidence (Cutler et al. 1990). Sixteen percent of participants reported incorrect PEI in their free recall as compared to 33% in the cued recall. This supports current investigative interviewing guidelines (Crown Prosecution Service 2011) which advise that witnesses, regardless of intoxication, should be questioned using free recall approaches designed to reduce the likelihood of externally introduced false information decreasing testimony accuracy.

In practical terms, because a witness could be influenced by both accurate and erroneous co-witness information, it may be favourable to prohibit all co-witness discussions. As alcohol does not seem to increase the reporting of misinformation from a co-witness, this advice should be followed regardless of the intoxication status of the witness.

## Footnotes

1 Participants’ responses were scored as correct if they were an accurate representation of the video. If participants used terms that were synonymous with those in the scoring sheet, this would be classed as correct.
